# REGULATORY COMPLIANCE AND RESIDENT OUTCOMES IN OREGON ASSISTED LIVING COMMUNITIES

**DOI:** 10.1093/geroni/igac059.2365

**Published:** 2022-12-20

**Authors:** Ozcan Tunalilar, Sunny Lin, Paula Carder

**Affiliations:** Portland State University, Portland, Oregon, United States; Washington University School of Medicine in St. Louis, St. Louis, Missouri, United States; Portland State University, Portland, Oregon, United States

## Abstract

The outsized negative impact of the COVID-19 pandemic among residents of assisted living (AL) communities has drawn attention to the existing challenges that licensing agencies face in providing oversight in this setting. While regulatory compliance inspections in AL may be a critical tool for promoting high-quality care, no published research has examined the association between deficiencies and resident outcomes in AL. Using novel data collected from 331 AL communities in Oregon (response rate=62%) merged with deficiency data from inspections, we examined whether the number of deficiencies is associated with the following resident outcomes as measured at the community level: share of residents who fell at least one time (fall rate), who were hospitalized overnight (hospitalization rate), and who were treated in the hospital emergency room (ER rate) in the last 90 days. Negative binomial regression models that controlled for a robust set of community (e.g., size, rurality, profit status, “memory care” units) and resident (e.g., age, Medicaid use, ADL needs) characteristics showed a weak, but statistically significant, association between deficiencies and hospitalization rate (IRR=1.05). However, there was no statistically significant association between deficiencies and falls or ER visits among residents. The time lag between inspections and the resident outcome data did not moderate these observed associations. With more states moving to make available such deficiency data on public-facing websites, these results have policy implications for the role and content of regulatory compliance inspections for ensuring quality in AL communities and informing the public.

